# EXPECTANT MANAGEMENT OR CHOLECYSTECTOMY IN ASYMPTOMATIC CHOLELITHIASIS

**DOI:** 10.1590/0102-6720202400047e1841

**Published:** 2024-12-02

**Authors:** Eduardo Neubarth TRINDADE, Lucas dos Santos DIFANTE, Luiz Roberto Rigo WENDT, Manoel Roberto Maciel TRINDADE

**Affiliations:** 1 Universidade Federal do Rio Grande do Sul, Hospital de Clínicas de Porto Alegre, Department of Surgery, Digestive Surgery Service - Porto Alegre (RS), Brazil

It is essential to reevaluate old concepts that, over time, become unquestionable truths. This is particularly important in the treatment of asymptomatic cholelithiasis, where an expectant, non-surgical approach is commonly recommended. We should therefore commend and congratulate the authors of the article “ASYMPTOMATIC CHOLELITHIASIS: EXPECTANT OR CHOLECYSTECTOMY. A SYSTEMATIC REVIEW,” published in the Brazilian Archives of Digestive Surgery (Arq Bras Cir Dig. 2023;36:e1747), for promoting the discussion of one of the most prevalent digestive disorders in the adult population.

Although it is a controversial topic in clinical practice, we agree with the authors’ conclusion that “the majority of evidence points to the safety and feasibility of a conservative (clinical follow-up) management of asymptomatic cholelithiasis”^1^. However, we need to consider that some variables are not always taken into consideration in systematic reviews, which can prejudice obtaining more robust conclusions.

It is important to note that there are no clinical trials directly comparing surgical and expectant treatments for asymptomatic cholelithiasis. In most cases, prophylactic cholecystectomy is not recommended due to the low risk of serious complications and the generally mild initial symptoms. The evidence supporting this recommendation, however, largely originates from studies conducted in the 1970s and 1980s, before the advent of laparoscopic surgery. For instance, in 1983, Ransohoff et al.^7^ concluded that conventional prophylactic cholecystectomy decreased patient survival based on a model analyzing the natural history of cholelithiasis. This type of study is now considered outdated considering the advancements in surgical techniques.

The standardization of laparoscopic surgery has drastically reduced the risks and complications associated with cholecystectomy, making it the prime example of the benefits of minimally invasive surgical techniques. Currently, serious complications of laparoscopic cholecystectomy, such as bile duct injuries, bleeding, and inadvertent bowel injuries, have an incidence of less than 0.5%. Surgical wound infection rates are also significantly lower compared to the open technique^4,9^. Additionally, the “critical view of safety” approach has been an ally in reducing harm and training new surgeons^2^.

The advantages of laparoscopic cholecystectomy over the open procedure have been further substantiated by a recent systematic review conducted by Roy et al.^8^ The authors demonstrated that patients undergoing laparoscopic cholecystectomy had notable reductions in mortality (odds ratio [OR] 0.30), mean hospital stay duration (mean difference: -2.68 days), major complications (OR 0.35), postoperative wound infections (OR 0.29), and duration of sick leave (OR 0.34) compared to those undergoing open interventions. While some studies in their selection noted a slightly higher incidence of bile leakage in the laparoscopic technique group, these findings, along with those concerning common bile duct injury, were not statistically significant. Overall, the results emphasize the superior safety and efficiency of laparoscopic cholecystectomy, highlighting its advantages over open procedures for managing gallbladder disease.

Furthermore, regional and temporal factors influencing outcomes are often overlooked in systematic reviews. Given that acute cholecystitis can be the first clinical manifestation of gallstone disease, it is important to consider the challenges in accessing the healthcare system in a country dependent on the Unified Health System for surgeries. In Brazil, it is worth considering the potential benefits of performing laparoscopic cholecystectomy in asymptomatic patients to prevent future complications. Laparoscopic cholecystectomy continues to be the gold standard treatment even in emergency situations for acute cholecystitis and can be safely performed in most patients, as highlighted by Coelho et al.^3^ However, a small group of high-risk patients, primarily the elderly with severe comorbidities, may not benefit from the laparoscopic approach. The higher rate of conversion to open surgery in emergency cholecystectomies, particularly in those with chronic cholecystitis, also warrants careful consideration. Performing laparoscopic cholecystectomy proactively in asymptomatic patients with gallstones may help alleviate these complications and improve patient outcomes.

Additionally, the diagnosis and evaluation of cholelithiasis symptoms are challenging, as they are often subjective and difficult to quantify. Atypical symptoms are more common than typical cholelithiasis symptoms. In cases of asymptomatic cholelithiasis where an expectant approach is chosen, it is important to remember that patients should be educated to recognize warning signs and seek medical attention before complications arise. This kind of understanding could be difficult to achieve in a country with low educational levels and insufficient healthcare infrastructure in various regions.

As the population’s life expectancy increases, we also need to consider the heightened potential for the development of biliary tract cancer (BTC) in individuals with gallstone disease, as recently identified by Huang et al.^5^ in a systematic review and meta-analysis. The analysis found that the presence of gallstones increases the risk of BTCs, with a notable OR of 7.26 for gallbladder cancer (GBC), 3.17 for extrahepatic bile duct cancer, and 3.28 for ampulla of Vater cancer. Among the risk factors, gallstone size is particularly critical; larger stones (>1 cm) were associated with a significantly higher risk of GBC (OR, 1.88). The prophylactic removal of the gallbladder in patients with large asymptomatic stones can potentially mitigate the heightened cancer risk inherent to such gallstone characteristics. Although surgical decisions should weigh the risks and benefits carefully, particularly concerning surgery-related morbidity and healthcare costs, the ability to significantly reduce the risk of developing these highly fatal cancers argues strongly for the consideration of prophylactic laparoscopic cholecystectomy in selected patients.

Designing a systematic review of a disease that evaluates surgical interventions presents inherent challenges, especially when the studies included require over a decade of follow-up and aim to maintain low sample dropout rates. Ensuring that these studies neither underestimate nor overestimate long-term harmful effects is complex. Moreover, addressing publication biases is crucial, as studies with positive outcomes are often published more frequently than those with negative results, potentially leading to an overestimation of the adverse effects of an intervention.

In current medical practice, it is essential to personalize evaluations by considering the potential risks and benefits of an intervention, such as minimally invasive cholecystectomy, while also considering the patient’s personal preferences and circumstances^6^. Given the current evidence in the literature, it is important to adopt a flexible approach when considering surgical indications for patients with asymptomatic cholelithiasis. This flexibility ensures that decisions are patient-centered and evidence-based, optimizing healthcare outcomes.
